# The Effect of an Automated Mobile Patient Engagement Application on Emergency Department Revisits: Prospective Observational Study

**DOI:** 10.2196/17839

**Published:** 2021-12-13

**Authors:** Pothik Chatterjee, Adam M Beck, Jenna Ashley Levenson Brager, Daniel J Durand, Christopher R D'Adamo

**Affiliations:** 1 Innovation and Research Department LifeBridge Health Baltimore, MD United States; 2 Department of Family & Community Medicine University of Maryland School of Medicine Baltimore, MD United States

**Keywords:** patient engagement, value-based care, digital health, mobile app, automation, readmission, revisit, emergency department

## Abstract

**Background:**

Revisits within 30 days to an emergency department (ED), observation care unit, or inpatient setting following patient discharge continue to be a challenge, especially in urban settings. In addition to the consequences for the patient, these revisits have a negative impact on a health system’s finances in a value-based care or global budget environment. LifeBridge Health, a community health system in Maryland, United States, implemented an automated mobile patient engagement application as part of our enterprise-wide digital health strategy to improve patient engagement and reduce revisits to the ED.

**Objective:**

The aim of this paper was to evaluate the effectiveness of a customized automated digital patient engagement application (GetWell Loop) to reduce 30-day revisits after home discharge from an ED.

**Methods:**

The LifeBridge Health Innovation Department and ED staff from 2 participating health system hospitals collaborated with GetWellNetwork to customize their patient engagement application with automated check-in questions and other on-demand resources (eg, streaming content explaining aspects of self-care during COVID-19). An application link was emailed to adult patients discharged home from the ED. A study of ED visits for patients treated for general medicine and cardiology conditions between August 1, 2018, and July 31, 2019, was conducted using CRISP (Chesapeake Regional Information System for our Patients), Maryland’s state-designated health information exchange. We also used data within GetWell Loop (GetWellNetwork) to track patient activation and engagement. The primary outcome was the number of ED patients who experienced a 30-day revisit and who did or did not activate their GetWell Loop account. Secondary outcomes included the overall activation rate and the rate of engagement as measured by the number of logins, alerts, and comments generated by patients through the application. Bivariate analysis comparing outcomes among patients who activated the GetWell Loop application to patients who did not was conducted using the Fisher exact test. Multivariate logistic regression modeling with elastic net regularization was also performed to account for potential confounders and potential collinearity of covariates.

**Results:**

During this 1-year study, 1062 (27.4%) of 3866 of all emergency patients treated for general medicine or cardiology conditions, who received an invite to use the digital application, activated their account. The patients discharged from the ED, who were treated for general medicine conditions (n=2087) and who activated their GetWell Loop account, experienced a 30-day revisit rate of 17.3% (n=101) compared with 24.6% (n=369) for those who did not activate their account (*P<*.001). Of the patients treated for cardiology conditions (n=1779), 12.8% (n=61) of those who activated their GetWell account experienced a 30-day revisit compared with 17.7% (n=231) of those who did not activate their account (*P*=.01). The significance of these findings persisted after adjustment for confounding variables including age, race, sex, and payor in logistic regression modeling (adjusted odds ratio 0.75, 95% CI 0.62-0.92; *P*=.006).

**Conclusions:**

Our results suggest that a significant percentage of patients are willing to utilize a digital application following ED discharge to better engage in their own care, and that usage of such digital applications may significantly reduce 30-day revisit rates. LifeBridge Health’s experience demonstrates that health care systems can leverage automated mobile apps to improve patient engagement and successfully impact clinical outcomes at scale.

## Introduction

Patients who are more actively engaged in their health care experience are more likely to demonstrate better health outcomes and incur lower costs. However, encouraging patients to engage in their health care outside of the clinic can be a challenge. Follow-up communication and adherence to postdischarge instructions are often inconsistent, leading to care gaps and preventable readmissions [1-4].

Revisits within 30 days to an ED, observation care unit, or inpatient setting following patient discharge continues to be a challenge, especially in urban settings. In addition to the consequences for the patient, these revisits have a negative impact on a health care system’s finances in a value-based care or global budget environment [5,6].

Mobile apps can help physicians more effectively engage patients outside of acute care settings. Reed et al [2] reported that patients with diabetes and other multiple chronic conditions who connected to health resources via smartphones, tablets, or computers were more likely to be in regular touch with their primary care providers and less likely to be hospitalized. Other groups have demonstrated that offering consumers secure messaging with their providers and the ability to make appointments and view their lab results on mobile devices all led to greater engagement levels [7,8].

Our primary study objective was to evaluate the effectiveness of GetWell Loop to reduce 30-day revisits after home discharge from the emergency department (ED) setting. This study was conducted by the Emergency and Innovation departments of LifeBridge Health, a community health system in Baltimore, Maryland, and implemented at 2 of our hospital EDs—Sinai Hospital of Baltimore and Northwest Hospital Center. Our secondary objectives were to assess patients’ willingness to adopt and utilize this technology. Our target adoption rate for the GetWell Loop application was 25% based on the previous efforts by the LifeBridge Health clinical call center as well as the activation and utilization rates of GetWell Loop at other health systems [9-12].

This study examined a digital health intervention to promote continuous patient support and aimed at reducing the number of revisits to the emergency room following discharge. The intervention consisted of a customized version of an already-existing patient engagement application, which was offered to all patients discharged from 2 LifeBridge Health emergency departments. Our hypothesis was that the use of an automated digital patient engagement application would significantly reduce ED revisits as a result of improved patient engagement and education, as well as more frequent check-ins and alerts. GetWell Loop prompts the user to log in to the application to answer questions, receive information, and view content at designated time periods following a specific event. These features are programmed ahead of time and are unique to each individual clinical event. For our study, the clinical event of interest was discharge to home following an ED outpatient encounter.

## Methods

### Design

The intervention studied was a web-based application prompting patients to “check in” for a defined period of time after a visit to the ED. The study population comprised adult patients who returned home from Sinai or Northwest hospitals after an outpatient ED visit related to a well-defined set of conditions described below. The specific web application studied was GetWell Loop. An interdisciplinary team, including both clinical and administrative staff and leaders from Sinai and Northwest hospitals, created an “ED Discharge Care Plan,” which included a specific set of questions, resources, and a checklist.

**Figure 1:**
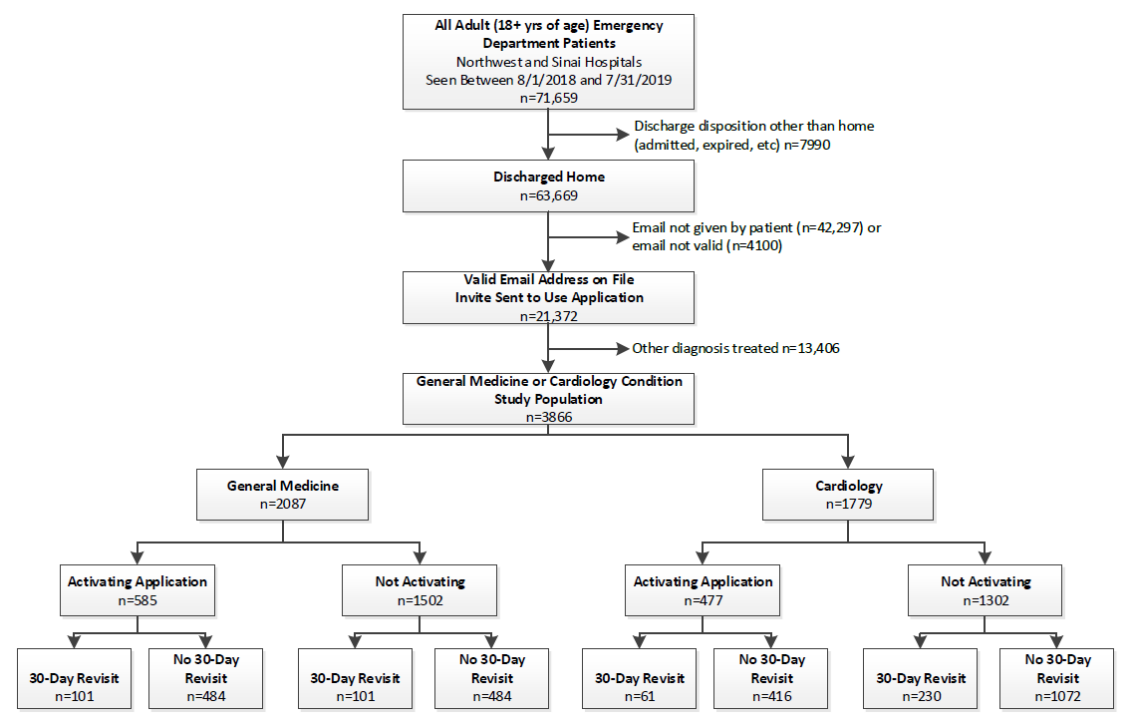
Population enrolled on the GetWell Loop application and focus population studied.

All patients discharged directly home after an ED visit were eligible to use the platform regardless of the reason for treatment. Therefore, the components of the study intervention were designed to apply to all patients treated in the ED with a focus on primary care, given that nearly 25% of the patients treated in the Sinai and Northwest EDs were treated for general medicine and cardiology conditions. To account for this focus, as well as to control for any impact due to a patient’s underlying medical condition, the study analysis was limited to those patients treated for general medical and cardiology conditions. In addition, those whose visit led to an inpatient admission were also excluded from this study, as additional modules and content were often added to the intervention in those cases that might further confound the analysis. Figure 1 represents the population enrolled and the specific population analyzed in this study.

The team identified on which postdischarge day each component of the intervention should be delivered to the patient (the cadence), using the discharge date as a reference date (Table 1). The “ED Discharge Care Plan” was designed to stay active for 5 days postdischarge. GetWellNetwork support staff assisted in customizing their software to embed the “ED Discharge Care Plan” in their GetWell Loop platform and automate the delivery of the questions and content through their application. The GetWellNetwork did not fund this study nor were its staff used for any follow up with patients engaging through the platform.

**Table 1:** Check-in questions, resources, and checklists with associated cadence included in the “ED Discharge Care Plan.”

Prompts, resources, and checklists			Days after discharge the questions were scheduled to be sent to the patient
**Check-in questions**			
	Welcome message after ED^a^ discharge	1	
	If you were given a prescription, do you have any questions about how to take your medications, such as which pills to take or how many times a day?	1	
	If you were given a prescription to fill, have you been able to fill it?	1	
	Since you visited the emergency department, do you feel your main problem has improved, stayed the same, or worsened?	1	
	Do you have any questions about your home care instructions?	1	
	Do you have a follow-up appointment?	1	
	Do you have any questions about the discharge instructions you received?	1	
	Please tell us how satisfied you were with your recent LifeBridge hospital visit?	2	
	How satisfied are you with using this application?	5	
**Resources**			
	Concerning symptoms after ED visit	1	
	Managing your follow-up appointments	1	
	Taking charge of your medications	1	
**Checklists**			
	Pick up prescriptions	1	

^a^ED: emergency department.

Each question was standardized with a specific answer choice, and each answer choice was identified by the implementation team to trigger a yellow alert, red alert, or no alert. The application also allowed patients to submit free text comments and questions along with answers to standardized questions. All alerts and comments were highlighted in real time by the GetWell Loop software through a clinical dashboard that was monitored during business days and business hours by agents at LifeBridge Health’s clinical call center. Yellow alerts were acted upon within 1 business day, red alerts were handled within an hour, and free-text comments and questions were prioritized individually after triage by the clinical call center staff. The agents in the call center followed up with patients addressing their needs and concerns with a primary focus towards connecting patients with community resources (ie, primary care physician, community pharmacist, etc). Communication with the patient was made either directly through the application or by telephone.

An interface between LifeBridge Health’s electronic health record (EHR; Cerner) and the application was implemented in order to auto-enroll patients on the application and initiate the “ED Discharge Care Plan.” Enrollment occurred immediately after the patient was discharged. Once enrolled, a user account for the GetWell Loop software was created for each patient, and an invite was emailed the following morning at 5 AM EST, prompting the patient to activate their account and verify their identity. Each patient was required to activate their account after enrollment in order to utilize the application and initiate the automated check-in process. The GetWell Loop software is available to the patient as both a web-based application and as a formal app that may be downloaded on a smartphone.

Once the patient activated their account, the “ED Discharge Care Plan” was initiated, and the first set of check-in questions was presented. The patients continued to receive emails autogenerated by the software to check in based on the configured intervals for each question. Reminder emails were submitted to patients who had not activated their account or missed a check-in. The application is HIPAA (Health Insurance Portability and Accountability Act)-secure and is tied to the patient’s EHR number. All communication activity documented by either a care provider or patient within the application was interfaced to the EHR and recorded within the patient’s formal electronic medical record.

### Eligibility Criteria

Adult patients (aged 18+ years) discharged home, with a valid email address entered in their record in the EHR, were enrolled. There were no exclusions made based on diagnosis for treatment, patient secondary diagnoses, or any other factor. Patients with an invalid email address or with “bounce backs” to the system were excluded from the study.

### Program Start

The patients were enrolled starting July 18, 2018, and continued through July 31, 2019. All ED staff including physicians, nurses, and registrars were educated with a focus on email collection and informing the patient at discharge. Brochures regarding the software were made available in the ED waiting areas, and a digital poster describing the program was included on all digital displays in the ED.

### Data Extraction and Measurement

The primary intervention was measured by the activation rate defined as the percentage of those invited who activated their account. This information was captured within the GetWell Loop platform.

To analyze the impact on 30-day revisits, CRISP (Chesapeake Regional Information System for our Patients), Maryland’s state-designated health information exchange was leveraged. The CRISP database was used to identify those ED visits in which a study patient experienced an emergency department visit, inpatient admission, or observation stay at any Maryland facility within 30 days of discharge. The CRISP database does not include visits to a primary care practice or urgent care facilities. The CRISP reporting system also provided diagnostic category groupings for each ED visit, which allowed the identification of each general medicine and cardiology encounter for analysis. CRISP uses 3M’s proprietary Enhanced Ambulatory Patient Grouping System to perform this task.

Information from the EHR was combined with the GetWell and CRISP data. These elements included patients’ age, sex, race, primary insurance, and primary diagnosis. The patients’ primary insurance was grouped according to the State of Maryland’s Health Services Cost Review Commission requirements (ie, Medicare, Medicaid, Medicaid Managed Care, etc).

The date range used for analysis was the 1-year period beginning on August 1, 2018, and ending on July 31, 2019. Any patient who was discharged home and experienced a visit to a Maryland emergency department within 30 days of discharge or who was admitted as an inpatient or observation patient to any Maryland hospital within 30 days of discharge was considered to have experienced a 30-day revisit. The intervention group was defined as those patients who activated their GetWell Loop account and initiated the “ED Discharge Care Plan,” regardless of whether they finished all of the modules and regardless of the degree of engagement in the platform.

### Statistical Analysis

Descriptive statistics were computed to characterize the study sample on key demographic characteristics including age, race, sex, and payor status. Bivariate analysis evaluating unadjusted associations between activation of the app and revisit rate was estimated utilizing a Wald chi-square test and a Fisher exact test. Multivariate logistic regression models were also constructed to adjust for potential confounders including age, sex, race, payor status, visit type, and primary diagnosis condition. Elastic net regularization was applied to account for potential collinearity among covariates in the regression models. General medicine and cardiology cohorts were defined based upon the service line groupings in the CRISP data set. Statistical significance was defined as *P*<.05. Statistical analyses were conducted in SAS (version 9.4.1, SAS Institute Inc).

## Results

A total of 3866 patients treated for general medicine (n=2087) and cardiology conditions (n=1779) invited to use GetWell Loop were studied to assess the impact of the application on 30-day revisit rates (Table 2). Combined, this group of patients represented 3866 (22.4%) of the 17,272 total population invited to use the application and 1062 (24.5%) of the 4337 of the total population who activated their account.

A total of 577 general medicine patients (28% of the total general medicine patients) and 477 cardiology patients (27% of the total cardiology patients) activated their accounts. The average age, sex, and primary payor classification for the cardiology and general medicine patients invited to use the application are listed in Table 2. There was no age difference found between cardiology patients who did not activate their account and those who activated their account (*P*=.64). The age of general medicine patients who activated their account (mean 52 years, SD 18.2) was greater than the age of those who did not activate their account (mean 50 years, SD 18.8; *P*=.02). There was a significantly greater proportion of female patients activating their account in both cardiology and general medicine populations (*P*=.03 and *P*=.02, respectively). A lower proportion of patients with Medicaid insurance activated their account, and there was no difference found in the activation rate for Medicare patients. African American patients activated their account much less frequently (*P*<.001) while White patients activated their account much more frequently (*P*<.001).

**Table 2:** Descriptive statistics for cardiology and general medicine emergency department patients enrolled on GetWell Loop, comparing those activating their account to those not activating their account.

Patient characteristics			All patients or all conditions invited to use application		All patients or all conditions activating their account		Cardiology patients not activating their account		Cardiology patients activating their account		*P* value^a^		General medicine patients not activating their account		General medicine patients activating their account		*P* value^a^
Patients, n			17,272		4337		1302		477		—^b^		1502		585		—
Age (years), mean (SD)			45.1 (17.7)		45.7 (17.4)		49.5 (16.8)		49.1 (15.9)		.64		49.85 (18.8)		52.03 (18.2)		.02
Female, n (%)			11658 (67.5)		3145 (72.5)		830 (63.7)		330 (69.2)		.03		955 (63.6)		404 (69.1)		.02
**Race, n (%)**																	
	Black or African American	13430 (77.8)		3131 (72.2)		1039 (79.8)		349 (73.2)		.004		1135 (75.6)		390 (66.7)		<.001	
	White	3031 (17.5)		973 (22.4)		196 (15.1)		109 (22.9)		<.001		291 (19.4)		154 (26.3)		<.001	
	Multiple	267 (1.5)		78 (1.8)		21 (1.6)		7 (1.5)		.99		26 (1.7)		13 (2.2)		.47	
	Declined to answer or unknown	250 (1.4)		77 (1.8)		17 (1.3)		2 (0.4)		.12		28 (1.9)		13 (2.2)		.60	
	Asian	178 (1.0)		51 (1.2)		19 (1.5)		9 (1.8)		.52		15 (1.0)		11 (1.9)		.12	
	American Indian or Alaska Native	64 (0.4)		18 (0.4)		5 (0.4)		1 (0.2)		.99		2 (0.1)		3 (0.5)		.14	
	Native Hawaiian, other Pacific Islander	52 (0.3)		9 (0.2)		5 (0.4)		0 (0)		.99		5 (0.3)		1 (0.2)		.99	
**Payor, n (%)**																	
	Commercial or other payor	7538 (43.6)		1927 (44.4)		546 (41.9)		249 (52.2)		<.001		465 (31.0)		259 (44.3)		<.001	
	Medicaid payor	6141 (35.6)		1301 (30.0)		377 (29.0)		117 (24.5)		.07		500 (33.3)		130 (22.2)		<.001	
	Medicare payor	3593 (20.8)		849 (19.6)		311 (23.1)		96 (20.1)		.10		444 (29.6)		170 (29.1)		.82	

^a^*P* value calculated using the Welch 2-sample *t* test (age), chi-square test (sex), and Fisher exact test (payor, race).

^b^Not applicable.

A multivariate logistic regression model was constructed to assess the impact of the intervention while adjusting for age, sex, race, primary payor, and primary diagnosis condition (general medicine or cardiology). After adjustment for covariates, patients who activated the application were significantly less likely to have a 30-day revisit (odds ratio 0.75, 95% CI 0.62-0.92; *P*=.006).

Age, sex, and race were not found to have a significant impact on the 30-day revisit outcome. Medicaid and Medicare patients were more likely to have a 30-day revisit (*P*<.001 and *P*=.02, respectively). The patients treated for a cardiology condition compared with those treated for general medicine were found to be less likely to have a 30-day revisit rate (*P*<.001).

The 30-day revisit rate for the general medicine subgroup who activated their GetWell Loop account was 17.3% (n=101) compared to 24.6% (n=369) of those who did not activate their account (*P*<.001; Table 3). For cardiology patients who activated their account, 12.8% experienced a 30-day revisit compared to 17.7% (n=231) of those who did not activate their account (*P*=.01). The percentage of check-in questions triggering the alert were as follows: follow-up appointment assistance (48%, n=917), prescription fill assistance (16%, n=306), discharge instruction questions (12%, n=229), understanding of treatment plan (10%, n=191), health status worsening (9%, n=172), med instruction questions (5%, n=96).

**Table 3:** Number of patients (and the associated 30-day revisit rates) treated for a general medical or cardiology condition in the Emergency Department, who were invited to use the GetWell Loop application.

Application usage or nonusage		Revisit within 30 days of discharge home, n	No revisit within 30 days of discharge home, n	Total, n	30-day revisit rate (%)	*P* value	
**Use of the application: general medicine patients**							<.001
	Number of patients not activating their account	370	1132	1502	24.6		
	Number of patients activating their account	101	484	585	17.3		
	Total	471	1616	2087	22.6		
**Use of the application: cardiology patients**							.01
	Number of patients not activating their account	230	1072	1302	17.7		
	Number of patients activating their account	61	416	477	12.8		
	Total	291	1488	1779	22.6		

## Discussion

### Strengths

The study benefitted from the use of many specific EHR data fields that are required for registration or for state reporting. Age, sex, race, primary payor, discharge disposition, and principal diagnosis are required state reporting elements and are reviewed at registration and during coding and billing. The email field is a required field during registration and includes an option for “none” or “patient refused.” These fields have been required elements for many years, and there were no new fields introduced in the EHR for this study.

The study also benefitted from automated enrollment on the platform based on these data fields. We did not have to rely on human intervention to specifically identify subjects for the study, meaning that any data-entry error and bias was applied across all potential candidates. All patient participants were invited to use the application in the same manner regardless of age, race, or socioeconomic status. We also limited our analysis to those patients who provided a valid email and thus invited to use the application rather than including all adult patients who returned home from their ED visit.

Our organization’s participation in Maryland’s health information exchange (CRISP) along with all other health systems in the state provided a robust and uniquely comprehensive data set for 30-day revisits. For several years, CRISP has been used to develop a model that links patient activity across all hospitals and their associated inpatient, ED, and observation care units leveraged for the analysis.

### Limitations

There were several limitations to our study. Patient acuity, secondary diagnoses, and other comorbid and socioeconomic circumstances play a role in a given patient’s potential to revisit an ED or to be readmitted to a hospital within 30 days after discharge from an ED. The study controlled for acuity by analyzing only adult patients treated for similar conditions (cardiology and general medicine) with a similar discharge disposition (discharged home directly from the ED). Cardiology and general medicine conditions were based on clinical groupings, and there was no attempt to analyze per the 10th revision of the International Classification of Diseases or to factor in secondary conditions. In an effort to control for technology use by patients and their likelihood of using the application, the study focused only on those patients who were invited to use the application and thus had provided a valid email address at registration.

There was no analysis of or comparison to those patients who were not invited, nor was there any means to control for the patients’ access to the internet, a mobile device, or a computer that is required for using the application. During the study, there was only 1 known organization-wide effort that was introduced that might have impacted the results. The community care coordinators in the EDs at both hospitals in this intervention were engaging identified patients who were high utilizers of ED services and were following up with these patients by telephone after discharge to the community; this was a small percentage of the total ED population, and it is felt to be unlikely that this significantly impacted the results discussed above. Our study was not able to determine whether this subgroup was enrolled on the application, and if so, whether they activated their account. Our study was also not able to identify and control for any other characteristic differences between those patients who activated their account and those who did not, such as education, lifestyle, social determinants of health (housing, transportation, and substance abuse), and access to primary care providers.

### Conclusion

The study revealed a statistically significant association between the use of the digital application and a lower revisit rate after discharge home. These results indicate the potential value of digital health applications to improve 30-day revisit rates.

The relative 30% lower revisit rate across both general medical and cardiology conditions sends a strong signal that the adoption of digital patent engagement tools can improve specific population-health outcomes and warrants further analysis to control for potential selection bias and chronic or comorbid conditions that may have additional patient acuity, which in turn may have impacted this study. The results also demonstrate that a significant percentage of patients are willing to utilize web-based applications to proactively engage in their own care following discharge. LifeBridge Health’s experience demonstrates that health care systems can leverage automated mobile apps to improve patient engagement and successfully impact clinical outcomes at scale. Further research should focus on expanding clinical use cases, enhancing activation rates, and studying and addressing barriers to patient adoption (eg, the impact of social determinants of health) in order to ensure that these methods improve, rather than exacerbate, disparities in health outcomes.

## Abbreviations

CRISP: Chesapeake Regional Information System for our Patients
ED: emergency department
EHR: electronic health record
